# Clinical and hemodynamic features of acute pulmonary embolism patients diagnosed in cold weather predicts adverse clinical outcome

**DOI:** 10.3389/fcvm.2022.1055926

**Published:** 2022-11-10

**Authors:** Na Sun, Yiqiang Chen, Xichao Liang, Youli Fan, Ming Fang, Xuan Gao, Yongbing Wang, Yansheng Chen, Zhuozhong Wang, Bo Yu, Jinwei Tian, Bingxiang Wu

**Affiliations:** ^1^Department of Cardiology, The Second Affiliated Hospital of Harbin Medical University, Harbin, China; ^2^Key Laboratory of Myocardial Ischemia, Ministry Education, Harbin Medical University, Harbin, China; ^3^Department of Cardiology, The First Affiliated Hospital of Harbin Medical University, Harbin, China

**Keywords:** acute pulmonary embolism, cold weather, hemodynamic, clinical, outcome

## Abstract

**Background:**

Acute pulmonary embolism (APE) is associated with peak incidence and mortality rate in winter. The present study sought to characterize the clinical and hemodynamic features of cold weather on APE patients.

**Methods:**

All enrolled 224 APE patients underwent clinical and hemodynamic evaluation and baseline parameters were collected. Recruited patients were grouped by weather pattern on admission into cold and warm weather group. The correlation and prognostic values among cold weather and other variables were analyzed.

**Results:**

Compared to warm weather group, patients in cold weather group present with more severe cardiac function, with adverse WHO-functional class (*P* = 0.032) and higher NT-proBNP concentration [1,853.0 (398.0, 5,237.0) pg/ml vs. 847.5 (56.8, 3,090.5) pg/ml, *P* = 0.001]. The cold weather group also displayed much critical hemodynamic status and heavier thrombosis load, with higher mPAP (29.1 ± 11.2mmHg vs. 25.6 ± 14.2mmHg, *P* = 0.045), higher PVR [3.3 (1.7, 6.0) wood units vs. 1.8 (0.9, 3.8) wood units, *P* < 0.001], higher Miller index (21.4 ± 5.9 vs. 19.1 ± 8.0, *P* = 0.024), and higher D-dimer levels [2,172.0 (854.5, 3,072.5) mg/L vs. 1,094.5 (210.5, 2,914.5) mg/L, *P* = 0.008]. Besides, cold weather showed well correlation with the above variables. Survival analysis showed APE patients in cold weather had significantly higher clinical worsening event rate (*P* = 0.010) and could be an independent predictor of adverse clinical outcome in the multivariate analysis (HR 2.629; 95% CI 1.127, 6.135; *P* = 0.025).

**Conclusion:**

APE patients in cold weather were associated with thrombus overload, cardiac dysfunction, hemodynamic collapse and higher clinical worsening event rate. Cold weather proves to be an independent predictor of adverse clinical outcome.

## Introduction

Acute pulmonary embolism (APE), caused by thrombus occlusion of pulmonary arteries, is a life-threatening disease with high mortality and morbidity rates(1, 2). A growing body of evidence has reported that the onset rate and mortality rate from major cardiovascular diseases is influenced by seasonal variation and APE is of no exception (3–5). Several studies demonstrated a peak incidence and mortality rate of APE during winter season, conflicting results on the influence of weather on disease outcomes have been presented in literature (3, 4, 6, 7). However, all published studies were epidemiology investigations, and systemic clinical and hemodynamic features of APE were not reported. Our center is located in the northeast part of China, which is one of the coldest regions in China with high incidence of pulmonary vascular and cardiovascular disease. Meanwhile, our center proved to be one of the largest regional diagnosis and management center for pulmonary vascular disease, which undertake the majority of diagnosis and treatment of patients from surrounding areas with pulmonary vascular disease. Therefore, the patients recruited in the present study may provide the robust evidence to further investigate the clinical and hemodynamic features of cold weather effect on APE patients.

As indicated, there could be a connection between cold weather and morbidity as well as mortality rate of APE, though specific pathophysiological mechanisms are still under investigation (8–11). Several factors may potentially contribute to cold weather effect on the adverse clinical outcome of APE such as abnormal coagulation system, peripheral vasoconstriction, reduced activity, combined cardiovascular disease, et al., which favors prothrombotic changes, cardiac dysfunction and hemodynamic collapse (6, 12, 13). However, previous studies failed to comprehensively investigate the cardiac function and hemodynamic status of APE patients and their values in predicting clinical outcomes.

In patients with APE, rapid occlusion of the vast of cross-sectional area of the pulmonary arteries may cause abrupt hemodynamic collapse, therefore, right ventricular function and hemodynamic compromise are vital determinant of long-term survival (14). Although the thrombus load, hemodynamic status and cardiac function in APE are decisive factors of mortality, their relationship with cold weather are still unknown. In present study, we consider to investigate the correlation among cold weather and thrombus load, hemodynamic parameters, cardiac function and clinical outcome in patients with APE. The present study aims to illustrate the clinical and hemodynamic features of APE patients in severe cold weather and to provide evidences of cold weather effect on clinical outcome of APE patients.

## Materials and methods

### Study design and patient population

A total of 262 APE patients were retrospectively screened at The Second Affiliated Hospital of Harbin Medical University from February 4, 2016 to December 17, 2020. Among them, 38 patients failed to participate in our study for the reason of either not tolerating invasive pulmonary angiography and right heart catheterization or failed to give informed consent. Therefore, a total of 224 adult patients with APE were finally recruited in our research. The inclusion criterions were as follows: (1) Ages 18–75 years; (2) Confirmed diagnosis of APE were made through computed tomography pulmonary angiography and/or pulmonary angiography following the updated guidelines (15, 16). Hemodynamic parameters were acquired from right heart catheterization and pulmonary angiography, which got informed consent from recruited patients. Patients who could not tolerate the invasive procedures and patients with pregnancy or chronic kidney disease were excluded from the study.

Upon diagnosis, baseline characteristics including history, demographic and clinical information, blood tests, echocardiography and hemodynamic parameters were collected. We also collected risk factor for APE patients. Risk factors are divided as DVT and others. For other risk factor in present study includes fracture, cancer, surgery, infection, et al. Enrolled patients were divided into two groups according to the local seasonal and air temperature pattern, the cold weather group (from January to March, and from October to December) and the warm weather group (from April to September). The treatment strategies for recruited patients were performed according to the international guidelines (17).

All recruited APE patients were followed up after discharge and the median follow-up time was 1.8 years. The major endpoint was the time to first clinical worsening event, defined as all-cause mortality and re-hospitalization for clinical worsening APE. Clinical worsening APE includes recurrent APE, deteriorating cardiac function and any other APE-related worsening event that need hospitalization. Four methods were adopted for patients follow up. First, recruited patients were followed up by phone calls. Second, APE patients were followed up by outpatient service. Third, enrolled patients were followed up by online outpatient service. Last, for the hospitalized patients, follow up information was recorded by review of medical record. The study was approved by the Ethics Committee of the Second Affiliated Hospital of Harbin Medical University. Besides, the study protocol conforms to the ethical guidelines of the Declaration of Helsinki. All enrolled patients gave their written informed consent.

### Hemodynamic measurement

Pulmonary angiography and right heart catheterization were performed in all enrolled patients with written informed consent. For pulmonary angiography, contrast medium was injected through a side hole of angiographic catheter into the main pulmonary artery by high pressure syringe and angiograms were reviewed together with chest x-ray, thereafter, APE was diagnosed according to the presence of obstructions and/or filling defects (18). Miller index was measured as previously reported (19).

Right heart catheterization was conducted by Swan-Gans catheter in all recruited patients during hospitalization. Hemodynamic parameters including mean pulmonary arterial pressure (mPAP), mean right atrial pressure (mRAP), pulmonary vascular resistance (PVR), cardiac output (CO) and cardiac index (CI) were measured in all enrolled patients. CO was measured in triplicate by the thermodilution technique with ice-cold isotonic saline solution. PVR was calculated using the equation PVR = Pulmonary arterial mean pressure (PAMP)—Pulmonary capillary wedge pressure (PCWP), that is trans-pulmonary gradient, which were divided by CO as previously described (20).

### Statistical analysis

For continuous variables with a normal distribution, it was expressed as mean ± standard deviation. For variables that were not normally distributed, data were expressed as numbers, percentages, and medians with the corresponding 25 and 75th percentiles (interquartile range). To describe the results for categorical parameters, the group-specific number and percentage of subjects in each category will be presented. Demographic, clinical features and hemodynamic parameters of patients subgrouped by weather pattern were compared by the Chi-square test, Mann–Whitney U test, or *t*-test, as appropriate.

According to the monthly average temperatures, the 12 months of each year were divided into four hierarchical variables. July to September, with the highest temperature was defined as number 1, accordingly, April to June was defined as number 2, October to December was defined as number 3, and January to March represent the lowest temperature was defined as number 4. The above temperature related hierarchical variables were compared with clinical and hemodynamic parameters of APE patients, accordingly Spearman's rank correlation method was conducted. To investigate the prognostic significance and independent association between cold weather effects on clinical outcome events, a two-step survival analysis was conducted (20). First, to estimate the hazard ratios (HRs) and 95% confidence intervals (CIs) for the association between covariates and outcomes, the univariate Cox proportional regression analysis was performed. In the second step, a multivariate Cox regression model was used to estimate the hazard ratios and 95% confidence intervals for the association between cold weather and outcome adjusted for the variables significant in the univariate analysis. A *P*-value of <0.05 was considered significant.

## Results

### Regional air temperature pattern and patients distribution

**Figure 1:**
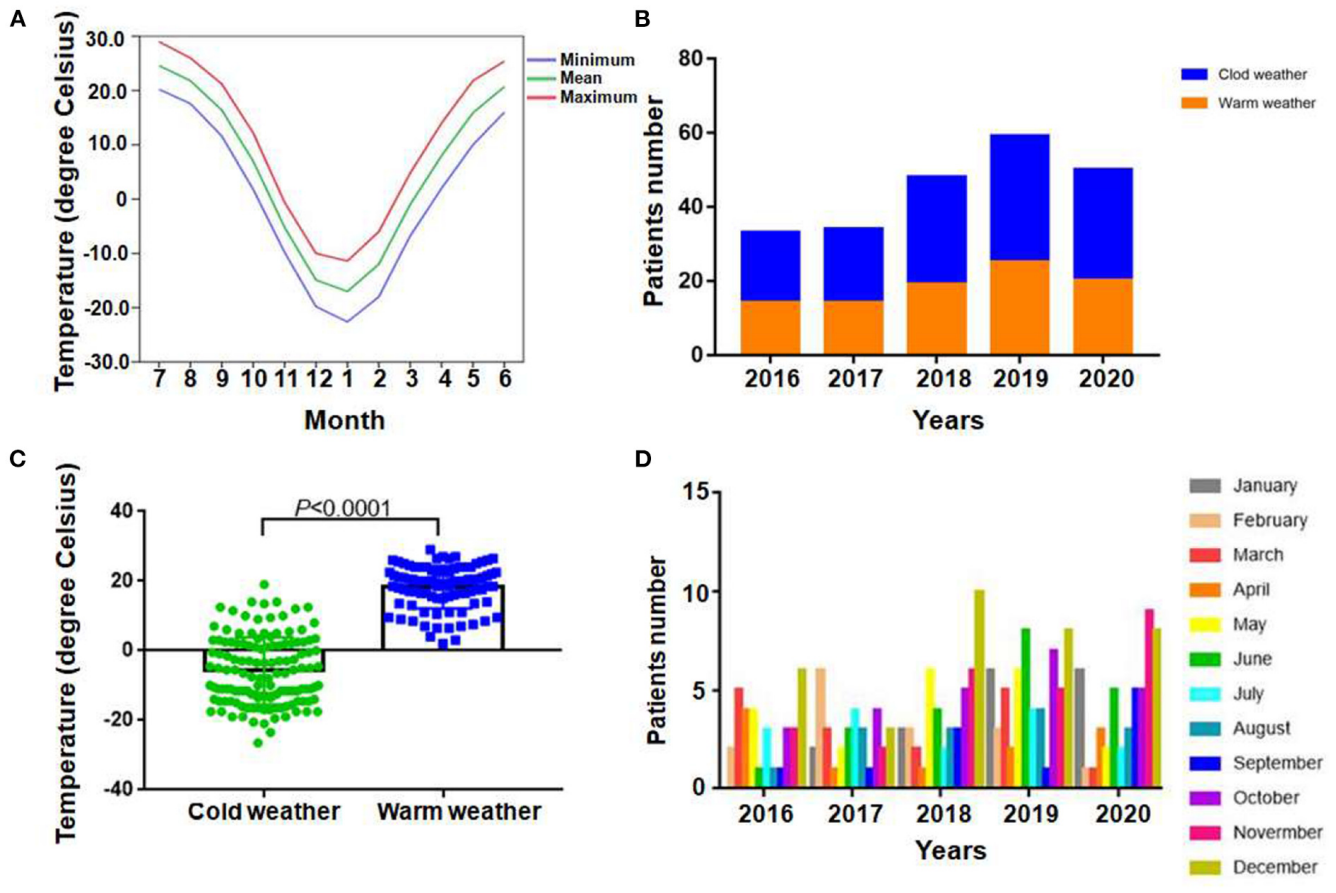
Regional air temperature patterns and distribution of patients. **(A)** Average monthly temperature distribution of enrolled patients locally from 2016 to 2020. **(B)** Total numbers of APE patients admitted during cold and warm weather in each year among 2016 to 2020. **(C)** Comparison between actual temperature of patients admitted in each group (*P* < 0.0001). **(D)** Detailed distribution of patients monthly.

For the study area, there are 6 months per year with the average air temperature near or below zero Celsius degree, the average temperature of the rest months are above zero Celsius degree (Figure 1A). Therefore, the recruited patients were divided into two groups according to the local temperature pattern, the cold weather group (from January to March, and from October to December) and the warm weather group (from April to September). From 2016 to 2020 years, the number of recruited APE patients was 33, 34, 48, 59 and 50, respectively. During study period, more APE patients were diagnosed and admitted during cold weather than warm weather each year (Figure 1B). Therefore the absolute patients number were much higher in cold weather group (132 vs. 92), in total 5 years. In addition, the evident difference of the actual temperature of the day patients admitted in the two groups were also observed (Figure 1C, *P* < 0.0001). The results demonstrated the high prevalence of APE in severe cold weather. The detailed enrolled patients' distribution monthly was presented in Figure 1D.

### Clinical characteristics of study population

Clinical characteristics are presented in Table 1. A total of 224 APE patients were recruited in our study, with 132 patients in cold weather group and 92 in warm weather group. Clinical parameters were compared between the two subgroups. Cold weather group showed more adverse cardiac function compared with warm weather group, with more adverse WHO-functional class level (*P* = 0.032), higher NT-proBNP concentration [1,853.0 (398.0, 5,237.0) pg/ml vs. 847.5 (56.8, 3,090.5) pg/ml, *P* = 0.001]. Furthermore, compared with warm weather group, cold weather group showed higher D-dimer levels [2,172.0 (854.5, 3,072.5) mg/L vs. 1,094.5 (210.5, 2,914.5) mg/L, *P* = 0.008], which indicate imbalanced fibrinolytic system. Also, the cold weather group presented lower PO_2_ (61.0 ± 23.0mmHg vs. 67.8 ± 21.8mmHg, *P* = 0.031), higher pericardial effusion rate (*P* = 0.021) and more combined PAH rate (*P* = 0.044), which were related to the severity of APE.

**Table 1:** Baseline clinical characteristics of study population in relation to cold weather.

	**Warm weather**	**Cold weather**	***P* Value^a^**
	**(*n =* 92)**	**(*n =* 132)**	
Age, years	60.5 ± 11.6	60.0 ± 12.2	0.799
Female gender, *n* (%)	34 (37.0)	63 (47.7)	0.110
BMI, (kg/m^2^)	25.3 .10.7	24.1 .10.4	0.247
WHO functional class, *n* (%)			0.032
II	53 (57.6)	55 (41.7)	
III	27 (29.3)	55 (41.7)	
IV	12 (13.0)	22 (16.7)	
RVD, mm	26.2 ± 7.6	22.8 ± 6.2	0.182
PO_2_, mmHg	67.8 ± 21.8	61.0 ± 23.0	0.031
TnI, μg/ml	0.04 (0.01, 0.20)	0.09 (0.02, 0.42)	0.197
NT-proBNP, pg/ml	847.5 (56.8, 3,090.5)	1,853.0 (398.0, 5,237.0)	0.001
D-dimer, mg/L	1,094.5 (210.5, 2,914.5)	2,172.0 (854.5, 3,072.5)	0.008
ALT, U/L	20.0 (14.0, 31.0)	23.0 (14.5, 42.5)	0.064
BUN, mmol/L	6.5 ± 3.0	7.0 ± 3.6	0.290
Creatinine, μmol/L	94.0 ± 36.5	90.5 ± 39.9	0.512
Uric acid, μmol/L	396.8 ± 139.2	365.5 ± 136.7	0.100
Pericardial effusion, *n* (%)	12 (13.0)	34 (25.8)	0.021
Risk factors, *n* (%)			0.714
DVT	16 (17.4)	26 (19.7)	
Others	21 (22.8)	31 (23.5)	
Combined PAH, *n* (%)	38 (41.3)	74 (56.1)	0.044
Reperfusion therapy, *n* (%)			0.405
Thrombolysis	8 (8.7)	17 (12.9)	
Interventional therapy	7 (7.6)	13 (9.8)	

Values are expressed as means ± SD, medians (interquartile range) or n(%).

RVD, right ventricular diameter; TnI, troponin I; PO_2_, oxygen pressure; NT-proBNP, N-terminal pro-brain natriuretic peptide; ALT, alanine aminotransferase; BUN, blood urea nitrogen.

a Comparison between warm weather group and cold weather group.

### Hemodynamic features of study population

Hemodynamic features are showed in Table 2. For all recruited patients, hemodynamic parameters were compared between cold weather group and warm weather group. Compared with warm weather group, cold weather group patients showed much heavier thrombus load manifested as higher Miller index (21.4 ± 5.9 vs. 19.1 ± 8.0, *P* = 0.024). In addition, the cold weather group present with hemodynamic collapse, with higher heart rate (93.8 ± 17.6 vs. 88.6 ± 18.2, *P* = 0.033), higher mPAP (29.1 ± 11.2mmHg vs. 25.6 ± 14.2mmHg, *P* = 0.045) and higher PVR [3.3 (1.7, 6.0) wood units vs. 1.8 (0.9, 3.8) wood units, *P* < 0.001].

**Table 2:** Hemodynamic features of study population in relation to cold weather.

	**Warm weather**	**Cold weather**	***P*-Value^a^**
	**(*n =* 92)**	**(*n =* 132)**	
HR, bpm	88.6 . 18.2	93.8 . 17.6	0.033
SBP, mmHg	122.3 2.22.2	126.3 6.23.8	0.213
Miller Index	19.1 .ex.0	21.4 .ex.9	0.024
mPAP, mmHg	25.6 .g,4.2	29.1 .g,1.2	0.045
mRAP, mmHg	6.9 Hg,.3	7.1 Hg,.8	0.728
PVR(Woods units)	1.8 (0.9, 3.8)	3.3 (1.7, 6.0)	< 0.001
CO (L/min)	5.3 /mi.7	4.9 /mi.5	0.134
CI (L/min/m^2^)	2.9 /mi.9	2.8 /mi.0	0.857

Values are expressed as means ± SD, medians (interquartile range) or n (%).

HR, heart rate; SBP, systolic blood pressure; mPAP, mean pulmonary artery pressure; mRAP, mean right atrial pressure; PVR, pulmonary vascular resistance; CO, cardiac output; CI, cardiac index.

a Comparison between warm weather group and cold weather group.

### Correlation between cold weather and other variables

The correlation analysis between cold weather and other variables are showed in Table 3. NT-proBNP (*R* = 0.213, *P* = 0.001), heart rate (*R* = 0.144, *P* = 0.033), D-dimer (*R* = 0.182, *P* = 0.008), Miller index (*R* = 0.161, *P* = 0.019), mPAP (*R* = 0.137, *P* = 0.045) and PVR (*R* = 0.256, *P* < 0.001) were positively correlated with cold weather. However, PO_2_ (*R* = −0.146, *P* = 0.031) were negatively correlated with cold weather. In addition, the variables associated with disease severity like pericardial effusion (*R* = 0.155, *P* = 0.020), WHO functional class (*R* = 0.144, *P* = 0.032) and combined PAH (*R* = 0.138, *P* = 0.044) were all correlated well with cold weather. Other variables failed to achieve statistical significance.

**Table 3:** Correlation of low temperature with clinical and hemodynamic parameters in APE patients.

**Parameters**	**R-value**	***P*-value**
NT-proBNP, pg/ml	0.213	0.001
PO_2_, mmHg	−0.146	0.031
HR, bpm	0.144	0.033
D-dimer, mg/L	0.182	0.008
Miller index	0.161	0.019
mPAP, mmHg	0.137	0.045
mRAP, mmHg	0.025	0.728
CO, L·min^−1^	−0.106	0.134
CI, L·min^−1^·m^−2^	−0.014	0.857
PVR, Woods units	0.256	<0.001
Pericardial effusion	0.155	0.020
WHO functional class	0.144	0.032
Thrombolysis therapy	0.065	0.330
Combined PAH	0.138	0.044

NT-proBNP, N-terminal pro-brain natriuretic peptide; PO_2_, oxygen pressure; HR, heart rate; mPAP, mean pulmonary arterial pressure; mRAP, mean right atrial pressure; CO, cardiac output; CI, Cardiac index; PVR, pulmonary vascular resistance.

### Survival analysis

**Figure 2:**
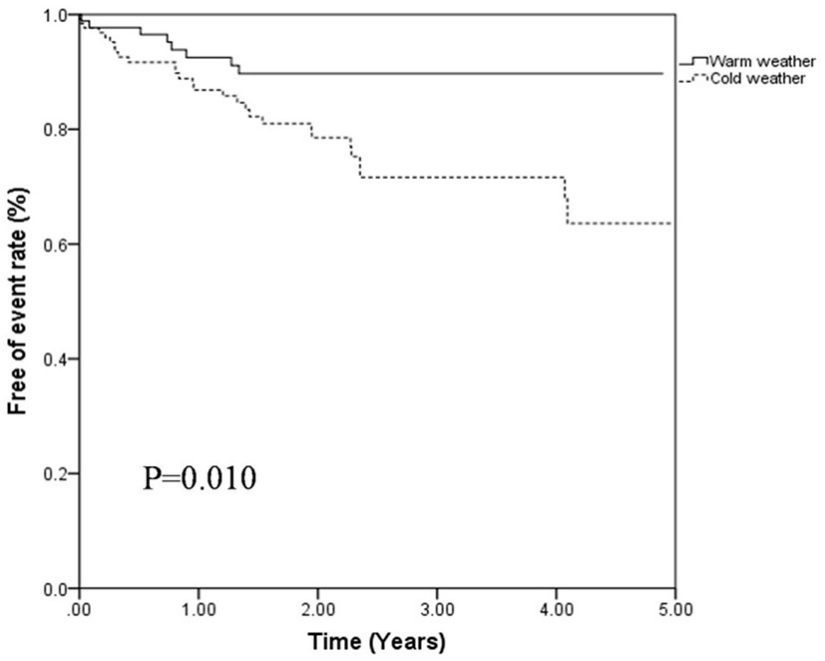
Free of events rates of patients with APE classified by cold weather group and warm weather group (*P* = 0.010, log-rant test).

During the follow-up period, 36 patients experienced clinical worsening events (16.1%), including 28 in cold weather group (21.2%) and 8 in warm weather group (8.7%). For the clinical worsening events in cold weather group, 8 patients died, 8 patients were hospitalized for recurrent thrombosis related events and 12 patients were hospitalized for aggravated cardiac function. In the warm weather group, clinical worsening events were comprised of 2 patients died, 3 patients hospitalized for recurrent thrombosis related events and 3 patients were re-hospitalized for aggravated cardiac function. Furthermore, during follow-up period, according to echocardiography and related clinical parameters, 6 (6.5%) patients progressed to CTEPH in warm weather group and 9 (6.8%) in cold weather group. In addition, 9 patients were lost for follow up, and their data were censored at the time of the last follow-up. Kaplan–Meier survival analysis demonstrated that APE patients in cold weather group had a significantly higher clinical worsening event rate (*P* = 0.010, Figure 2).

In the univariate Cox proportional regression analysis, cold weather (*P* = 0.046), peripheral effusion (*P* = 0.020), reperfusion therapy (*P* = 0.032) and mPAP (*P* = 0.013) were significantly associated with the risk of clinical worsening event. In the multivariate Cox regression analysis, cold weather (HR 2.629; 95% CI 1.127, 6.135; *P* = 0.025) and mPAP (HR 1.035; 95% CI 1.010, 1.061; *P* = 0.005) were associated with a higher risk of clinical worsening event after adjustment by age, sex and other variables significant in the univariate analysis as showed in Table 4. Thus, APE patients admitted in cold weather are more likely to experience clinical worsening event than those in warm weather.

**Table 4:** Univariate and multivariate analysis of risk factors in patients with APE.

**Variables**	**Univariate analysis**		**Multivariate analysis**	
	**HR[95%CI]**	***P-*value**	**HR[95%CI]**	***P*-value**
Age, years	1.012 (0.977, 1.049)	0.501	–	NS
Sex	0.939 (0.377, 2.330)	0.889	–	NS
Cold weather	2.632 (1.010, 6.797)	0.046	2.629 (1.127, 6.135)	0.025
HR, bpm	1.023 (0.998, 1.048)	0.072	–	NS
WHO functional class	1.030 (0.899, 1.055)	0.101	–	NS
NT-proBNP, pg/ml	1.189 (0.365, 2.010)	0.395	–	NS
D-dimer, mg/L	1.010 (0.895, 1.915)	0.078	–	NS
Miller index	1.066 (0.988, 1.151)	0.097	–	NS
mPAP, mmHg	1.057 (1.012, 1.105)	0.013	1.035 (1.010, 1.061)	0.005
PVR, Woods units	0.931 (0.806, 1.075)	0.128	–	NS
Reperfusion therapy	1.913 (1.260, 2.903)	0.032	–	NS
Pericardial effusion	2.215 (1.132, 4.333)	0.020	–	NS

NT-proBNP, N-terminal pro-brain natriuretic peptide; mPAP, mean pulmonary arterial pressure; PVR, pulmonary vascular resistance.

## Discussion

As a life threatening disease, APE contributes to the main cause of mortality and morbidity worldwide (21–23). The comprehensive influence of cold weather on APE is still not fully understood. In present study, considering the overall climate background may better affect clinical character of APE patients, therefore, we grouped patients according to cold and warm weather group instead of actual temperature of patients on the day of admission. Several major findings were as follows: (1) More APE patients were admitted and diagnosed during cold weather than warm weather group. (2) APE patients in cold weather group had more adverse cardiac function, much heavier thrombus load and hemodynamic collapse at the time of hospitalization. (3) Cold weather correlated well with thrombus load (Miller index), cardiac function (WHO functional class, NT-proBNP), hemodynamic parameters (heart rate, mPAP, PVR) and abnormal coagulation and fibrinolytic system (D-dimer) in patients with APE; (4) Follow-up analysis demonstrated that APE patients in cold weather had a significantly higher clinical worsening event rate and could be an independent predictor of clinical outcome.

Several factors may play a role in the pathophysiological process of APE patients in cold weather. First, cold weather may induce abnormal coagulation process and contribute to thrombus overload. In cold weather, the cooling surface of the skin and the tissues beneath it may promote an increased blood viscosity and imbalance release of certain factors leading to the changes in coagulation, which induce the formation of thrombus (3, 24–26). Also, the hypercoagulable status could be aggravated by reduced patient activity in cold weather, which cause the reduction of blood flow and increase the thrombosis formation (25, 27). As found in our study, APE patients in cold weather may present higher Miller index and D-dimer value, which well confirmed thrombus overload. Second, cold weather could aggravate hemodynamic compromise in patients with APE. Low temperature is accompanied by an increase in sympathetic tone, blood pressure, heart rate and myocardial oxygen consumption, which all leading to the pulmonary vascular bed collapse (6, 8, 9). To make it worse, the thrombus occlusion of pulmonary artery may cause the abrupt increase in mPAP and PVR that leading to right ventricular failure, which is the major cause of death from APE (28, 29). The current study just provided evidence for the above hypothesis that the enrolled patients in cold weather present with higher mPAP and PVR. Likewise, hemodynamic compromise plays a vital role in the mortality of APE patients, as Martin et al. (18), indicated in a series of 76 patients with APE, long-term prognosis was related to the level of mPAP and the presence of right ventricular failure. Third, in cold weather, the accompanied hemodynamic collapse could result in cardiac dysfunction. The sudden rise in mPAP and PVR abruptly elevated right ventricle afterload, which caused muscle stretch and increased wall tension leading to right ventricle dilation and dysfunction (30, 31). The low temperature further promotes the myocardial oxygen consumption, which increases the risk of cardiac adaptations (6). Our investigation further enhanced the above points with the cardiac function parameters of WHO-functional class and NT-proBNP much higher in cold weather group, however, the right ventricle diameter between the two subgroups failed to reach statistical significance. Finally, cold weather may predict poor prognosis in patients with APE. Reidel et al. (18) considered that the prognosis of APE can be predicted by PAP and right ventricle function at the time of diagnosis of the first episode of APE. Similarly, Toro et al. (32) and Olié et al. (33), indicated that low temperature could predict higher incidence and mortality rates in PE patients, which are consistent with our results. However, the previous research failed to illustrate the potential clinical reasons for the poor prognosis of patients with APE admitted in cold weather. In contrast, our results indicated that the thrombus overload, cardiac dysfunction and hemodynamic collapse may partially explicit the adverse clinical outcome of APE patients admitted in cold weather.

In present study, pulmonary angiography and right heart catheterization were performed in all recruited patients to fully investigate the effect of cold weather influence on thrombus load and hemodynamic parameters in patients with APE, therefore, Miller index was selected as the assessment method for thrombus load. Miller index, as a traditional parameter to reflect thrombus load, was achieved through pulmonary angiographic process, which could better indicate the authentic status of thrombus load (19). In addition, it is our major strength to conduct the accurate and comprehensive hemodynamic evaluation of cold weather influence on patients with APE. As a result, we discovered the thrombus load, hemodynamic parameters and cardiac function were closely related to each other, which makes it more reliable to detect the cold weather effect on patients with APE.

There are several limitations of the present study. First, the hemodynamic data of recruited patients were collected only at baseline, and the repeated clinical and hemodynamic parameters were not collected during follow-up period. In addition, the present study is a single center investigation with limited sample size, and several confounding factors also influence the further accurate evaluation of cold weather effects on aggravation of APE, therefore, a randomized, multicenter study with large sample size may needed to further testify the present results. Moreover, the present study is a retrospective study and failed to involve the risk stratification in the analysis, and further study focus on correlation between specific risk stratification and cold weather are needed. Finally, the present study failed to explore the potential molecular mechanisms from the aspects of cellular and animal model.

In conclusion, APE patients diagnosed in cold weather had a significantly higher clinical worsening event rate and could be an independent predictor of clinical outcome in multivariate analysis, which were probably due to the more severe cardiac dysfunction, thrombus overload and hemodynamic collapse at the time of hospitalization.

## Data availability statement

The raw data supporting the conclusions of this article will be made available by the corresponding authors at reasonable request, without undue reservation.

## Ethics statement

The studies involving human participants were reviewed and approved by Ethics Committee of the Second Affiliated Hospital of Harbin Medical University. The patients/participants provided their written informed consent to participate in this study.

## Author contributions

NS contributed to the study design, data acquisition, and data analysis and manuscript preparation. YiC and XL contributed to data analysis and manuscript preparation. ZW and BY contributed to the data analysis. YW, MF, and YaC contributed to the data acquisition. XG and YF contributed to the measurement of Miller index. JT and BW contributed to the study design and revision of the manuscript revision. All authors have reviewed the manuscript and approved the final version for publication.

## Funding

This work was supported by grants from the National Natural Science Foundation of China (Grant Nos. 81870043, 82270049 to BW and 81971715, U21A20391 to JT), the Fok Ying-Tong Education Foundation for Young Teachers, China (Grant No. 171032 to JT), the HMU Marshal Initiative Funding (Grant No. HMUMIF-21020 to JT), and Key Laboratory of Myocardial Ischemia, Harbin Medical University, Chinese Ministry of Education (Grant No. KF202007 to NS).

## Conflict of interest

The authors declare that the research was conducted in the absence of any commercial or financial relationships that could be construed as a potential conflict of interest.

## Publisher's note

All claims expressed in this article are solely those of the authors and do not necessarily represent those of their affiliated organizations, or those of the publisher, the editors and the reviewers. Any product that may be evaluated in this article, or claim that may be made by its manufacturer, is not guaranteed or endorsed by the publisher.
